# Comparison of chromosomal aberrations frequency and polymorphism of GSTs genes in workers occupationally exposed to cytostatics or anaesthetics

**DOI:** 10.2478/v10102-009-0016-0

**Published:** 2009-09-28

**Authors:** Ľudovít Mušák, Erika Halašová, Tatiana Matáková, Lucia Letková, Ludmila Vodičková, Janka Buchancová, Henrieta Hudečková, Oto Osina, Pavel Souček, Pavel Vodička

**Affiliations:** 1 Institute of Medical Biology, Comenius University in Bratislava, Jessenius Faculty of Medicine in Martin, Slovakia; 2 Clinic of Occupational Medicine and Toxicology, Comenius University in Bratislava, Jessenius Faculty of Medicine in Martin, Slovakia; 3 Institute of Medical Biochemistry, Comenius University in Bratislava, Jessenius Faculty of Medicine in Martin, Slovakia; 4 National Institute of Public Health in Prague, Czech Republic; 5 Institute of Experimental Medicine Academy of Sciences in Prague, Czech Republic; 6 Institute of Public Health, Comenius University in Bratislava, Jessenius Faculty of Medicine, Slovakia

**Keywords:** chromosomal aberrations, cytostatics, anaesthetics, occupational exposure, polymorphisms of GST genes

## Abstract

Authors compared the incidence of chromosomal aberrations (CAs) of workers occupationally exposed to cytostatics (group EXP1) or anaesthetics (group EXP2) in relationship to polymorphism of *GSTM1*, *GSTP1* and *GSTT1* genes. The cytogenetic analysis for chromosomal aberrations frequency and for polymorphisms of genes the PCR and PCR-RFLP method were used. Statistically higher frequency of total CAs was detected in both exposed groups: group EXP1 1.90±1.34%; Mann-Whitney U-test, *p*=0.001; group EXP2 2.53±1.46%, *p*=0.0008) as compared to control (1.26±0.93%). In group EXP2 was detected statistically higher frequency of aberrations CSA-type as compared to CTA-type. In xenobiotic metabolizing genes for GST higher frequency of total CAs and constituent types chromatid-type aberrations (CTAs) and chromosome-type aberrations (CSAs) of genes *GSTM1* and *GSTT1* with null genotype was detected. Statistically significant difference was detected only in CSA-type of aberrations in *GSTT1* gene. In gene *GSTP1* was not detected any difference in frequency of aberrations in presence of the variant allele. Presented results point out importance of individual susceptibility in evaluation of genotoxic agents of anaesthetics or cytostatics.

## Introduction

The mutagenic, carcinogenic and cytotoxic effect of cytostatics (IARC 1996, Burgaz *et al*., 2002, Cavallo *et al*., 2005; Cavallo *et al*., 2007; Rekhadevi *et al*., 2007; Poljaková *et al*., 2008) and anaesthetics (IARC 1996, Jaloczynski 1999, Karpinski 2005) is permanently discussed. Workers in oncological units are occupationally excessively exposed to low doses of cytostatics and anaesthesiologists to volatile anaesthetics in the long term. Cytostatics genotoxicity is expressed especially by undirected damage of genetic material. The most of mutagens are electrophilic agents, or can be metabolised by electrophilic intermediators. Connor (2006) reports that the skin is the first meeting point of antineoplastic agents′ contamination. After occupational exposure to cytostatics the increased frequency of chromosomal aberrations, sister chromatid exchanges (SCE) or micronuclei (MN) in cells was described by many authors (Pilger *et al*., 2000; Burgaz *et al*., 2002). Musak *et al*. (2006) published the higher frequency of CAs in variant type of alleles in DNA repair genes *XRCC1* exon 10 and *XRCC3* exon 7 and Mušák *et al*. (2008) detected statistically decrease of total CAs and CTAs in presence of variant allele. Testa *et al*. (2007) did not find out significant relationship between polymorphisms of genes for GST and higher frequency of CAs. Angelini *et al*., (2008) detected important influence of genotype *GSTT(–)*, in healthy individuals and after exposure to bleomycine (BLM). Rombaldi *et al*. (2009) analysed the genotoxicity using comet assay, and MN test as well as the level of oxidative stress. The exposed workers presented increased DNA damage levels by the comet assay as compared to the controls. The comet assay results have also shown significant positive correlation with the length of exposure and with the amount of consumed alcohol.

The anaesthesiologists are excessively occupationally exposed to volatile anaesthetics. The genotoxicity of desflurane was assessed by comet assay as extent of DNA fragmentation in peripheral human lymphocytes *in vitro*. The increased DNA migration was detected not only in cells exposed to halothane, but also to desflurane (Karpinski *et al*., 2005). There was not detected any increase of chromosomal aberrations′ number in patients exposed to anaesthetics during operation evidencing (Karahalil *et al*., 2005). Exposure to residues anaesthetics can increase the genetic damage comparable with damage caused by smoking 11–20 cigarettes per day (Hoerauf *et al*., 1999). The increased incidence of CAs was detected by Rozgaj *et al*. (1999) in medical workers in operating rooms. Rozgaj and Kasuba (2000) and Rozgaj *et al*. (2001) assessed frequencies of CA, MN and SCE in anaesthesiologists, and found their increase particularly in females. Bilban *et al*. (2005) found out increased frequency of CAs (2.69%) in medical workers of operating and reaniming rooms, which was comparable to group of radiologists. The increase of DNA damage was detected as well by Chandrasekhar *et al*. (2006) using comet assay, MN test and peripheral blood lymphocytes analysis in buccal mucosa cells. His findings were not dependent on age and gender of observed persons.

The aim of study is detection of genotoxic effect′s biomarkers – total CAs, chromatid-type (CTAs) and chromosome-type (CSAs) of chromosomal aberrations, and evaluation of individual susceptibility of *GSTM1*, *GSTP1* and *GSTT1* genes in medical workers occupationally exposed to cytostatics or anaesthetics.

## Material and methods

Seventy two workers (group EXP1) occupationally exposed to cytostatics and 76 workers (group EXP2) exposed to anaesthetics were analysed for frequency of chromosomal aberrations and polymorphisms of GST genes in comparison to control individuals. All of them completed anamnestic questionnaire about length and way of exposure, job categorize, exogenous factors (smoke, drug usage, exposure to radiation, alcohol consumption and dietary) before blood collection and give an approving to be involved in the study. Group EXP1 represented workers from specialized oncologic departments from three hospitals in north part middle Slovakia region – Faculty Hospital in Martin (n=28), Central Military Hospital in Ružomberok (n=31) and Hospital with Polyclinic in Trstená (n=13). All of workers were regularly in contact with cytostatics that dilute and apply to patients. By job grade they are classified as nurses (80.55%) and physicians. Both groups consist predominantly of females (90.28%). Smokers presented 26.39%. Group EXP2 consists of 76 workers from Faculty Hospital in Martin (n=60) and Central Military Hospital in Ružomberok (n=16). All of workers were regularly exposed to volatile anaesthetics. By job grade they are classified as anaesthesiologic physicians (53.95%) and nurses (46.05%). They participated in application of volatile anaesthetics during surgical interventions in complete anaesthesia in operating rooms. Groups consist predominantly of female persons (80.26%). Smokers presented 30.26%. Control group consisted of workers of Medical Faculty Hospital in Martin and workers from Biotika factory in Martin. They were not exposed to any genotoxic agents. Characteristics of both exposed groups and control are present in Table 1. We microscopically analysed 100 mitoses per person and evaluated the frequency of total chromosomal CAs, and constituent types: CTA-type and CSA-type of aberrations. Methodology of cytogenetic analysis was performed according to AHEM (2007). Polymorphisms of GST genes were performed by PCR methods. Gene *GSTM1* primers – F (forward): 5′-CTG CCC TAC TTG ATT GAT G-3′; R (reverse): 5′-CTG GAT TGT AGC AGA TCA TGC-3′; size of fragments: 1) normal homozygote or heterozygote (*GSTM1-plus*) – 275 bp (*GSTM1*) and 175 bp (*GSTM2*), 2) variant homozygote (*GSTM-null*) – *GSTM1* product not form and 175 bp (*GSTM2*). Genes *GSTP1* and *GSTT1* primers *GSTP1*-F (forward): 5′- TCC TTC CAC GCA CAT CCT CT-3′; *GSTP1*-R (reverse): 5′-AGC CCC TTT CTT TGT TCA GC-3′; *GSTT1*-F (forward): 5′-TTC CTT ACT GGT CCT CAC ATC TC-3′; *GSTT1*-R (reverse): TCA CCG GAT CAT GGC CAG CA; size of fragments 1) normal homozygote or heterozygote (*GSTT1-plus*) – 480 bp; 2) variant homozygote (*GSTM-null*) – product not form; restriction enzyme *BsmA*I, size of fragments: 1) normal homozygote (Ile105Ile) – 294 bp, 2) heterozygote (Ile105Val) – 294 + 234 + 60 bp, 3) variant homozygote (Val105Val) – 234 + 60 bp. All principles for protection personnel data, for health care are accepted in this paper. Peripheral blood sampling was realized within the specialised medical examinations. Statistical analysis was performed by program Statgraphics, version 7 (Manugistics, Cambridge, MA). There was used nonparametric Mann-Whitney U-test for testing differences between groups and analysis of variance (ANOVA) for testing relationships between biomarkers and genotype. The values in tables are as average±standard deviation (S.D.)

**Table 1:** Characteristics of exposed groups and control.

	EXP1	EXP2	Control
Number	72	76	76
Age (years±S.D.)	41.19±8.95	36.89±8.75	35.99±7.73
Exposure (years±S.D.)	11.31±8.98	11.75±9.35	
Sex (N) M/F	7 / 65	15 / 61	16 / 60
Smoking (N) S/NS	19 / 53	23 / 53	15 / 61
Job (N) physician/nurse	14 / 58	41 / 35	20 / 56

**Table 2:** Chromosomal aberrations expressed percentage in exposed groups and control.

	Number of mitosis (N)	Number of Ab.c. (N)	Percentage of CAs (%)±S.D.
EXP1 - cytostatics	7200	137	1.90±1.34^***^
EXP2 - anaesthetics	7600	192	2.53±1.46^***^
Control	7600	96	1.26±0.93

^***^ *p*=0.001 (EXP1)

^***^ *p*=0.0008 (EXP2)-total CAs exposed vs. control

## Results

**Figure 1:**
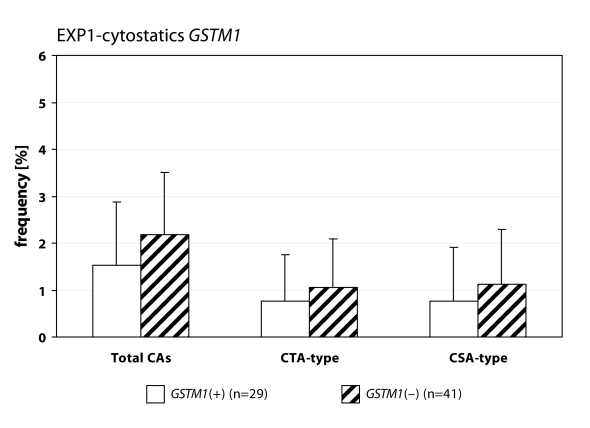
Polymorphisms of *GSTM1* gene in exposed group to cytostatics.

**Figure 2:**
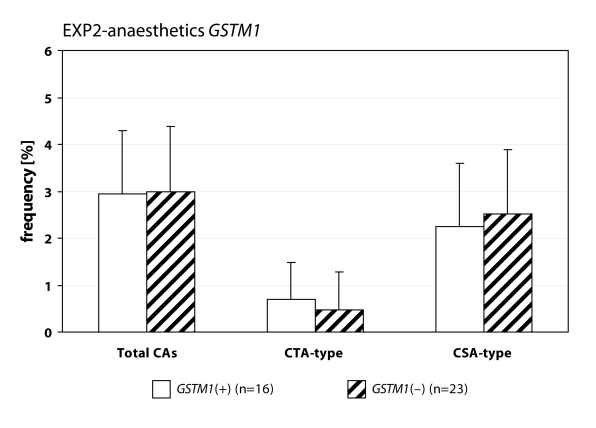
Polymorphisms of *GSTM1* gene in exposed group to anaesthetics.

**Figure 3:**
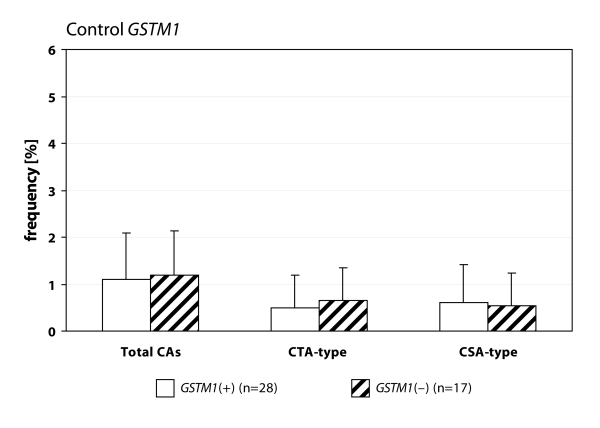
Polymorphisms of *GSTM1* gene in control group.

**Figure 4:**
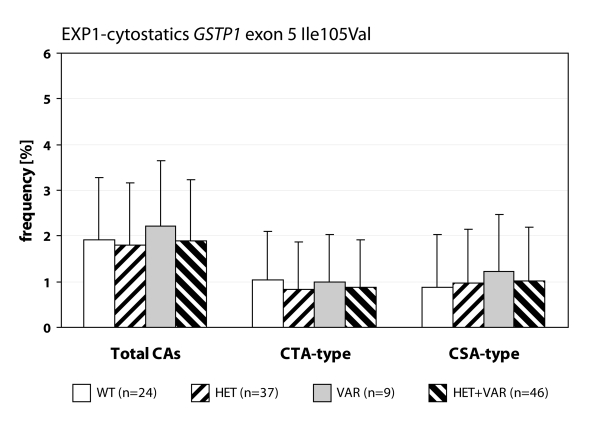
Polymorphisms of *GSTP1* gene in exposed group to cytostatics.

**Figure 5:**
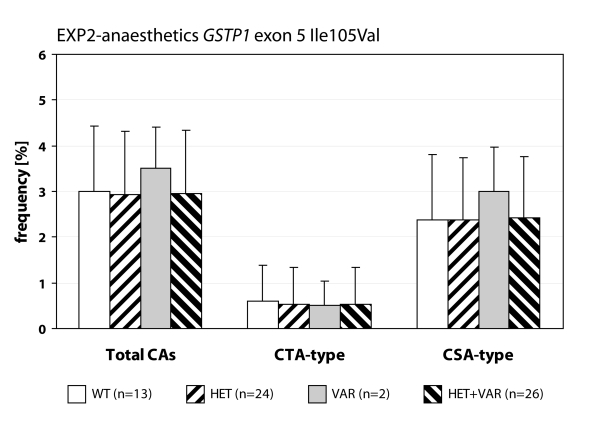
Polymorphisms of *GSTP1* gene in exposed group to anaesthetics.

**Figure 6:**
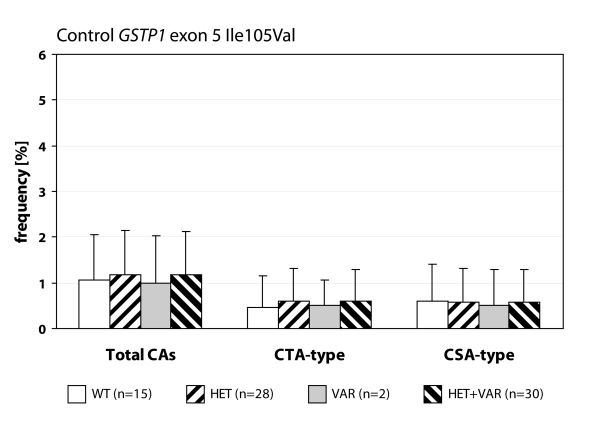
Polymorphisms of *GSTP1* gene in control group.

**Figure 7:**
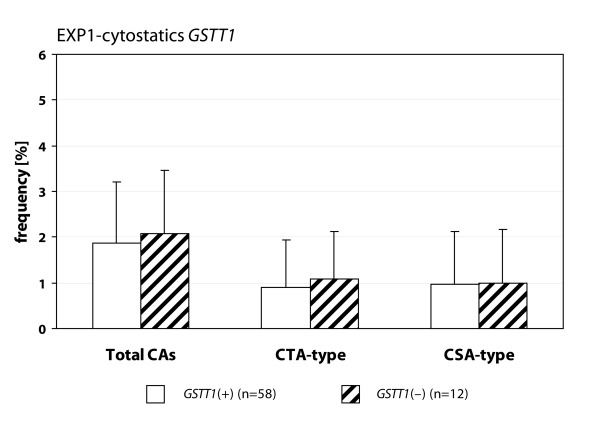
Polymorphisms of *GSTT1* gene in exposed group to cytostatics.

**Figure 8:**
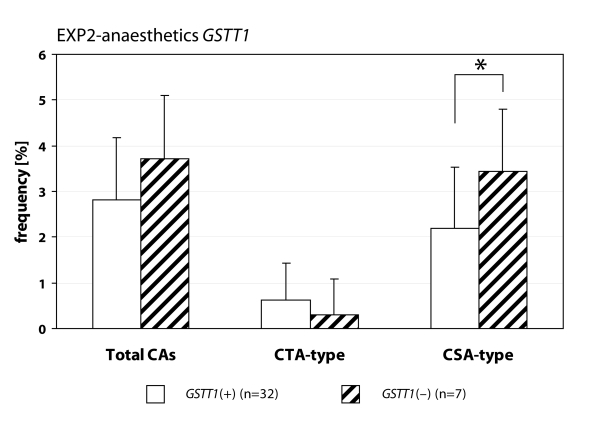
Polymorphisms of *GSTT1* gene in exposed group to anaesthetics.

**Figure 9:**
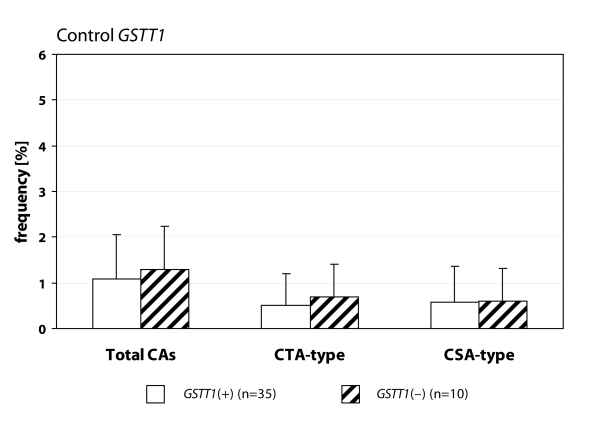
Polymorphisms of *GSTT1* gene in control group.

We detected statistically higher frequency of total chromosomal aberrations (CAs) in group EXP1 (1.90±1.34%; *p*=0.001) and group EXP2 (2.53±1.46%; *p*=0.0008) in comparison to control (1.26±0.93%). In group EXP1 we did not detect any difference between CTA-type and CSA-type – 0.92±1.04% vs. 0.98±1.17%; on the contrary in group EXP2 we detected statistically higher frequency of aberrations CSA-type in comparison to CTA-type (1.92±1.38% vs. 0.61±0.83%; *p*=0.0009). Evaluating the role of polymorphisms of *GSTM1* gene we did not observed significant difference in group EXP1, but in group EXP2 we detected the increase of total CAs, CTA-type and CSA-type in null variant as compared to plus variant (Figures 1, 2 and 3). Evaluating of *GSTP1* gene polymorphisms we did not detect any differences in presence of variant allele (Figures 4, 5 and 6). Evaluation of *GSTT1* gene showed slightly increase of total CAs, and CTA-type in group EXP1, and higher (statistically not significant) frequency of total CAs as well as significant difference (*p*=0.05) in null variant as compared to plus variant of CSA-type in group EXP2 (Figures 7, 8 and 9).

## Discussion

Cytostatics are mutagenic and carcinogenic agents (Vorlíček *et al*., 2000). They cause variously DNA damage. Burgaz *et al*. (2002) and Cavallo *et al*. (2005) examined workers in contact with cytostatics (nurses and pharmaceutical workers that prepared, diluted or applied cytostatics; orderlies in contact with bed sheets and possibly excrements of treated patients). They both observed the higher frequency of CAs and MN in buccal mucosa cells in exposed workers. Burgaz *et al*. (2002) detected 2.5-time higher frequency of CAs in exposed persons.

Anaesthesiology workers are notable exposed to volatile anaesthetics during occupational activity. In many papers is presented the increase of aberrant cells in operating rooms workers (Rozgaj *et al*., 1999; Rozgaj *et al*., 2001; Natarajan, 2002). Our findings are in accordance with previously mentioned; we detected statistically higher frequency of total CAs in group EXP1 and group EXP2 as well. Numerous authors determined in their explorations that the increased risk to exposure of genotoxic agents is related with inconsistent observance of safety regulations. The higher exposures to cytostatics were observed at workplaces, where the personnel was not adequately informed about risk associated with this job (Connor, 2006; Fransman *et al*., 2007a; Fransman *et al*., 2007b ). The increased number of chromosomal aberrations in group EXP2 can be caused by unsuitable conditions of employment (i.e. not effective or inadequately effective air circulation, operating rooms without air-conditioning) in comparison to control. The extensive research of employment conditions showed, that in operating rooms with 10–12 multiple air circulation only, can be the concentration of halothane decreased to 50–80 mg.m^–3^ (Wiesner *et al*., 2001). The role of gene-environmental interactions has been frequently discussed for last years and the research results have pointed on their relation to diseases formation in some individuals. It is believed that this interaction can be influenced by gene polymorphism (Horváthová, 2008). For the determination of CAs frequency in human lymphocytes we analysed specific polymorphisms of genes participated in metabolism of genotoxic agents. We analysed the polymorphism of GSTs genes. In gene *GSTM1* and *GSTT1* we detected higher frequency of total CAs and constituent types i.e. CTA-type and CSA-type in both exposed groups. Scarpato *et al*., (1997) detected the higher frequency of CAs in smokers in absence of allele *GSTM1* gene (*GSTM1 null variant*). The absence of gene for *GSTM1* and *GSTT1* point out the higher frequency of CAs in comparison with presence *GSTM1* gene and absence *GSTT1* gene in drivers of busse (Norppa *et al*., 2006).

Presented results finger on importance of individual susceptibility in assessment of genotoxic effects, in cases, the concentration of genotoxic agents usually doesn't exceed the occupational exposure limits.
